# Effect of Multiple-Nutrient Supplement on Muscle Damage, Liver, and Kidney Function After Exercising Under Heat: Based on a Pilot Study and a Randomised Controlled Trial

**DOI:** 10.3389/fnut.2021.740741

**Published:** 2021-12-23

**Authors:** Chunbo Wei, Shengnan Zhao, Yuntao Zhang, Wenbo Gu, Shuvan Kumar Sarker, Shuande Liu, Benzhang Li, Xuanyang Wang, Ying Li, Xu Wang

**Affiliations:** ^1^National Key Discipline, Department of Nutrition and Food Hygiene, School of Public Health, Harbin Medical University, Harbin, China; ^2^Department of Neurosurgery, The 962nd Hospital of the PLA Joint Logistic Support Force, Harbin, China

**Keywords:** hot environment, inflammatory reaction, oxidative stress, recovery, multiple nutrients

## Abstract

**Objective:** This study explored the effect of multiple-nutrient supplementation on muscle damage and liver and kidney function after vigorous exercise under heat.

**Methods:** After an initial pilot trial comprising 89 male participants, 85 participants were recruited and assigned into three groups: a multiple-nutrient (M) group, a glucose (G) group, and a water (W) group. Multiple-nutrient supplements contain glucose, fructose, maltose, sodium, potassium, vitamin B_1_, vitamin B_2_, vitamin C, vitamin K, and taurine. Participants were organised to take a 3-km running test (wet-bulb globe temperature 32°C) after a short-term (7 days) supplement. Blood samples were obtained to detect biochemical parameters [glucose (GLU), aspartate aminotransferase (AST), alanine aminotransferase (ALT), blood urea nitrogen (BUN), uric acid (UA), creatinine (Cr), creatine kinase (CK), lactate dehydrogenase (LDH), and lactic acid], inflammation factors [interleukin-6 (IL-6) and tumour necrosis factor-α (TNF-α)], and oxidative stress biomarkers [superoxide dismutase (SOD) and 8-iso-prostaglandin F (2alpha) (8-iso-PGF2α)].

**Results:** In the pilot trial, BUN decreased significantly in the M and G groups immediately after the running test. AST, Cr, and UA were significantly reduced 24 h after the running test with single-shot multiple-nutrient supplementation. In the short-term trial, multiple nutrients further prevented the elevation of CK (*p* = 0.045) and LDH (*p* = 0.033) levels 24 h after strenuous exercise. Moreover, we found that multiple nutrients significantly reduced IL-6 (*p* = 0.001) and TNF-α (*p* = 0.015) elevation immediately after exercise. Simultaneously, SOD elevation was significantly higher in the M group immediately after exercising than in the other two groups (*p* = 0.033). 8-iso-PGF2α was reduced in the M group 24 h after exercise (*p* = 0.036).

**Conclusions:** This study found that multiple-nutrient supplementation promoted the recovery of muscle damage and decreased liver and kidney function caused by strenuous exercise in a hot environment, probably through the inhibition of secondary damage induced by increased inflammatory reactions and oxidative stress. In this respect, the current study has important implications for the strategy of nutritional support to accelerate recovery and potentially prevent heat-related illness. This study was prospectively registered on clinicaltrials.gov on June 21, 2019 (ID: ChiCTR1900023988).

## Introduction

The rate of heat-related morbidity and mortality increases with the process of climate change, especially for workers and athletes (1–3). Exposure to a hot and humid environment increases metabolic rates and heat production. Meanwhile, body heat dissipation also increases, with increase in sweating and cutaneous vasodilation. Consequently, the cardiovascular system responds by increasing heart rate and cardiac contractility and reducing blood flow from non-cutaneous regions (4). A huge body of studies has concluded that high environmental temperature negatively affects the physical performance and exacerbates poor cognitive function (5–8). Beyond that, a hot environment augments immune disturbance (9–11) and oxidative stress (production of reactive oxygen species (ROS) and lipid peroxidation, etc.) (12, 13). On the whole, all the physiological mechanisms believed to cause injury with hot conditions alone are markedly aggravated further with physical activities (14–17).

However, most of the studies are conducted to provide nutrient supplements under thermal conditions. In addition, there is no conclusive instruction about the kind and amount of nutrients to consume. Accumulating evidence has proven that chronic exposure to a hot environment leads to nutrient deficiency. For instance, several minerals (such as sodium, potassium, calcium, magnesium, iron, and iodine) and water-soluble vitamins (vitamin B and vitamin C) were lost while sweating (18–21). Some scientists recommended that it is not necessary to consume nutrient supplements since they could be replenished if they follow a normal diet. Notwithstanding, researchers found nutritional supplements to be an ergogenic aid and to repress oxidant and inflammatory responses. Carbohydrate supplementation had a benefit on endurance (22) and anaerobic (23) performance. Supplementation with sodium helped in preventing heat cramping (24). Vitamin C supplementation decreased post-exercise cortisol in athletes anticipating marathons in a hot environment (25).

Moreover, not all studies concluded that nutrient supplementation was effective when a single nutrient was used. Actually, it is not an independent consequence of a single mechanism but the complex interaction of the entire body that responds to the physical strain caused by the combination of a hot environment and physical activities. Given the scarcity of literature concerning this, the current study aimed to explore the effect of multiple-nutrient supplement supplementation on strenuous activity in a hot environment and its advantage, if any, over glucose and water consumption.

## Methods

### Subjects

This study consisted of two randomised, single-blinded trials: a pilot trial (single-shot supplement trial) involving 89 male participants who completed a running test after a single-shot supplement; a second trial (short-term supplement trial) in which 90 participants were enrolled and in which 85 finally completed the running test after a 7-day short-term supplement supplementation (1 because of toothache, 2 got injured, and 2 caught flu). All subjects were between 18 and 32 years old, and all started to perform physical activities in hot conditions 1–15 years ago. All participants signed the written informed consent. The protocols of this study received approval from the 962nd Hospital of The PLA Joint Logistic Support Force and the Harbin Medical University Ethics Committee and conformed to the Helsinki Declaration for Human Research Ethics. This study was prospectively registered on clinicaltrials.gov (ID: ChiCTR1900023988).

### Nutritional Intervention

All participants in the above two trials were randomly assigned to three groups: the multiple-nutrient group (M group), glucose group (G group), and water group (W group). The components of the supplements in the current study are listed in Table 1. The dose of each component was determined according to former studies (26, 27) and the Chinese standard for sports nutritional food and functional food (GB/T 24154-2015). The multiple-nutrient supplement contained 6% carbohydrate, which was the main source of energy. Glucose provided energy rapidly. The proportion of glucose: fructose was 2:1. It was documented that the co-ingestion of fructose and glucose may increase the total carbohydrate absorption and oxidation (28). Maltose lowered the osmolality of the supplement. Sodium and potassium were added to balance the losses from sweat. Vitamin B helped in energy utilisation and vitamin C and taurine were antioxidant components. Furthermore, an extra 90 μg of vitamin K2 was specifically added to the short-term supplement to enhance the antioxidation and anti-inflammation capabilities. An equal concentration of carbohydrate control (6% glucose) was used in this study.

**Table 1:** Components and dosage of each shot.

	**Glucose**	**Single-shot multiple-nutrient supplement**	**Short-term multiple-nutrient supplement**
Glucose (g)	30.0	13.5	13.5
Fructose (g)	-	6.5	6.5
Maltose (g)	-	10.0	10.0
Sodium (mmol)	-	7.5	7.5
Potassium (mmol)	-	1.5	1.5
VB_1_ (mg)	-	3.0	3.0
VB_2_ (mg)	-	3.0	3.0
VC (mg)	-	200.0	200.0
VK (μg)	-	-	90.0
Taurine (g)	-	3.5	3.5
Energy (kJ/L)	501.6	472.4	472.4
Osmolality (mOsm/L)	333.3	311.3	311.3

*Each shot equals to 500 mL*.

### Experimental Design

**Figure 1:**
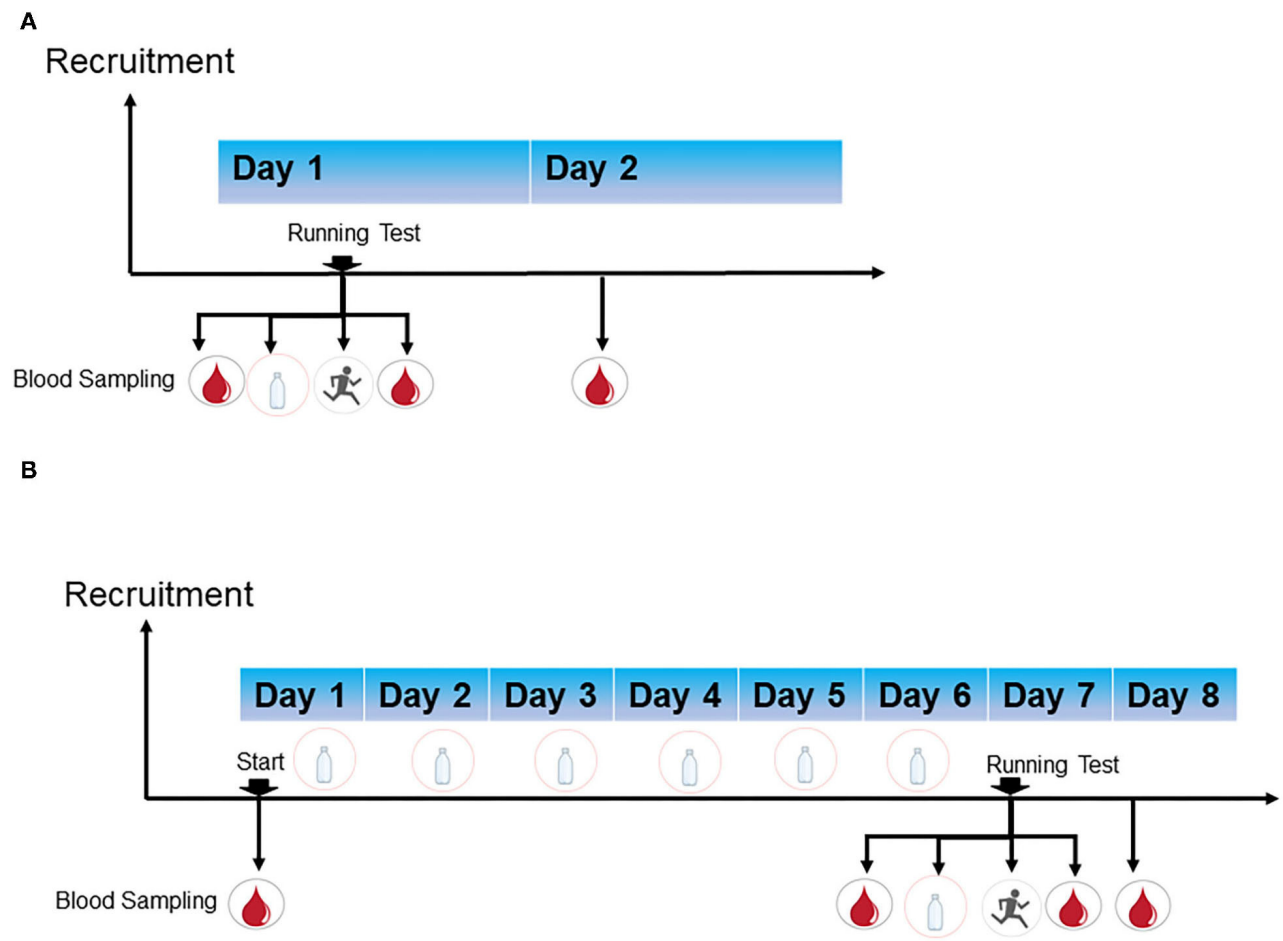
Time scheme of the single-shot supplement trial (pilot trial) **(A)** and short-term supplement trial **(B)**.

Two separated trials were conducted. Demographic information (such as age and length of work) and anthropometric parameters (height, weight, waist circumference, blood pressure, etc.) were collected at baseline (Tables 2, 3 and Supplementary Tables 1, 2). The time schedule of these two trials is graphically presented in Figure 1.

**Table 2:** Anthropometric characteristics of participants in short-term supplement trial at baseline.

	**Water group**	**Carbohydrate group**	**Supplement group**	***p***
N	27	30	28	
Age (y)	21.82, 0.42	22.73, 0.61	21.82, 0.41	0.650
Length of work (y)	3.37, 0.42	4.03, 0.62	2.93, 0.37	0.630
Height (cm)	175.11, 1.01	174.88, 1.13	176.66, 1.31	0.500
Weight (kg)	71.29, 2.12	71.82, 1.53	70.02, 1.86	0.577
Waist (cm)	78.12, 1.55	77.67, 1.56	75.44, 1.34	0.467
Body fat percentage (%)	17.44, 1.21	17.09, 0.95	15.31, 0.81	0.336
BMI (kg/m^2^)	23.23, 0.64	23.48, 0.45	22.10, 0.49	0.227
Systolic pressure (mmHg)	123.30, 2.42	125.63, 2.30	121.93, 2.91	0.591
Diastolic pressure (mmHg)	71.81, 1.88	69.87, 1.55	68.93, 1.97	0.528
Heart rate (bpm)	73.89, 1.95	74.41, 2.39	75.54, 1.56	0.843

*Data are presented as means ± standard error of the mean (SEM)*.

*BMI-body mass index*.

**Table 3:** Biochemical parameters of participants in short-term supplement trial at baseline.

	**Water group**	**Carbohydrate group**	**Supplement group**	***p***
AST (U/L)	24.96, 1.38	22.53, 1.18	26.29, 2.28	0.232
ALT (U/L)	20.30, 1.73	16.57, 1.44	22.25, 2.98	0.060
AST/ALT	1.37, 0.10	1.95, 0.45	1.35, 0.08	0.137
GLU (mmol/L)	4.31, 0.07	4.52, 0.14	4.34, 0.06	0.539
BUN (mmol/L)	5.22, 0.11	5.40, 0.22	5.32, 0.18	0.956
CREA (μmol/L)	80.19, 2.12	81.73, 1.98	78.39, 1.75	0.346
UA (μmol/L)	467.11, 19.66	419.53, 14.76	425.64, 17.12	0.113
CK (U/L)	369.70, 112.24	268.87, 54.88	436.29, 127.14	0.249
LDH (U/L)	202.67, 7.32	190.47, 5.15	202.89, 6.83	0.289
Na (mmol/L)	137.56, 0.26	137.40, 0.22	137.43, 0.16	0.760
K (mmol/L)	3.83, 0.06	3.78, 0.05	3.73, 0.07	0.510

*Data are presented as means ± standard error of the mean (SEM)*.

In the pilot trial, participants were required to arrive at the laboratory after overnight fasting. They were organised to take a 3-km running test 30 min after consuming 500 ml beverages in total according to their group (250 ml beverages each time according to their group, two times separated by 20 min). The Borg scale was used to assess the rating of perceived exertion (RPE) immediately after the running test. The two trials were conducted in summer and heat-related illness most easily occurred during this season. Wet-bulb globe temperature (WBGT) is an index that takes both temperature and humidity into consideration. The calculated WBGT was 26–27°C, and heat risk was a “high risk of heat injury” (29, 30).

In the short-term supplement, participants were arranged to consume the corresponding beverages on 7 consecutive days. After a period of 6 days of supplementation, they were required to arrive at the appointed place after overnight fasting on the 7th day to complete the final supplement. Then, they were organised to have a 3-km running test 30 min after consuming 500 m of the corresponding beverages in total (250 mL each time, separated by 20 min as described above). Trial time and RPE were recorded. The WBGT on the test day was 32°C, and heat risk was considered an “extreme risk of heat injury.”

Participants were gathered to have the same meals throughout the experimental period (from 3 days before the trial to the last blood sampling) to control for potential confounding factors of dietary intake. An extra 3-day period before the trial was called the equilibration period. They were instructed to refrain from consuming food and drink containing caffeine, taurine, alcohol to avoid strenuous exercise, and not to use anti-inflammatory medicine or massage throughout the trial.

### Blood Sampling and Parameter Analyses

Blood samples were collected from an antecubital vein of the same side before (PRE), immediately (POST), and 24 h after the running test (REC). All samples were allowed to clot and were centrifuged at 3,000 rpm/min for 15 min. Biochemical indices were determined using autobiochemical analysers (7100; Hitachi, Tokyo, Japan, for pilot trial, AU5800; Beckman, CA, USA, for the second trial). Parameters reflecting liver and renal function [serum aspartate aminotransferase (AST), alanine aminotransferase (ALT), glucose (GLU), blood urea nitrogen (BUN), creatinine (Cr), and uric acid (UA)], indirect markers of muscular damage [creatine kinase (CK) and lactate dehydrogenase (LDH)], blood electrolyte concentration [sodium (Na) and potassium (K)], lactic acid, inflammation markers [interleukin-6 (IL-6) and tumour necrosis factor-α (TNF-α)], and markers of oxidative stress [8-iso-prostaglandin F(2alpha) (8-iso-PGF2α) and superoxide dismutase (SOD)] were detected (31, 32). Lactic acid was detected utilising the colorimetric method (Nanjing Jiancheng Bioengineering Institute, Nanjing, China). Markers of inflammation and oxidative stress were measured using ELISA kits following the instructions of the manufacturer (Jiangsu Meimian Industrial Co., Ltd, China). The remaining blood samples were stored at −80°C for subsequent detection.

### Statistical Analysis

Variables in tables and figures are presented as the mean ± SEM. Changes in biochemical parameters immediately after the running test were calculated by POST minus PRE. Then, 24 h after running the test, changes were calculated by REC minus PRE. Normally distributed data were analysed using one-way ANOVA and skewed data (AST, ALT, AST/ALT, GLU, BUN, Cr, CK, and Na at baseline). AST and CK change at POST, and CK and LDH change at REC were analysed using the non-parametric tests. All analyses were conducted using IBM SPSS Statistics (version 21.0, IBM, NY, USA). A two-tale *p* ≤ 0.05 was defined as statistically significant.

## Results

### Effect of Multiple-Nutrient Supplementation on Serum Glucose and Lactic Acid

**Figure 2:**
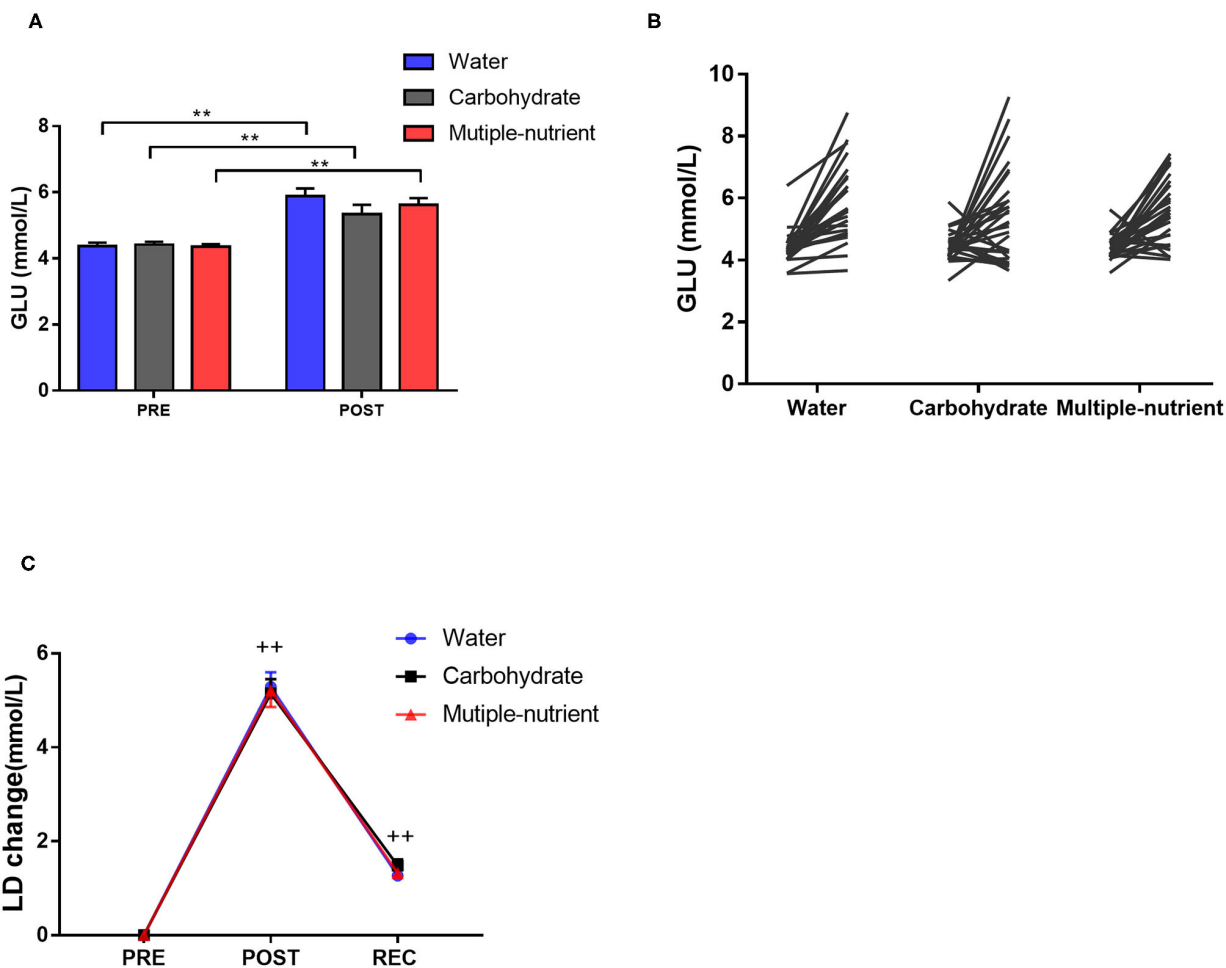
Fatigue-related parameters of each group in the short-term supplement trial. Glucose levels before and after running test **(A)**. Individual response of each group **(B)**. Change in lactic acid levels at different time points **(C)**. Values are mean ± SEM. PRE, prior running test; POST, immediately after running test. **Indicates *p* < 0.01. ++Indicates difference compared with PRE, *p* < 0.01.

Serum glucose increased significantly after the running test, with no difference among the groups at either PRE or POST (Figure 2). Lactic acid was significantly increased after the running test, while no significant difference was found among the groups regarding lactic acid change either at POST or REC. Consistent results were found in the single-shot supplement trial (Supplementary Figure 1).

### Multiple-Nutrient Supplementation Alleviated Heat-and Exercise-Related Muscle Damage and Liver and Kidney Injury

**Figure 3:**
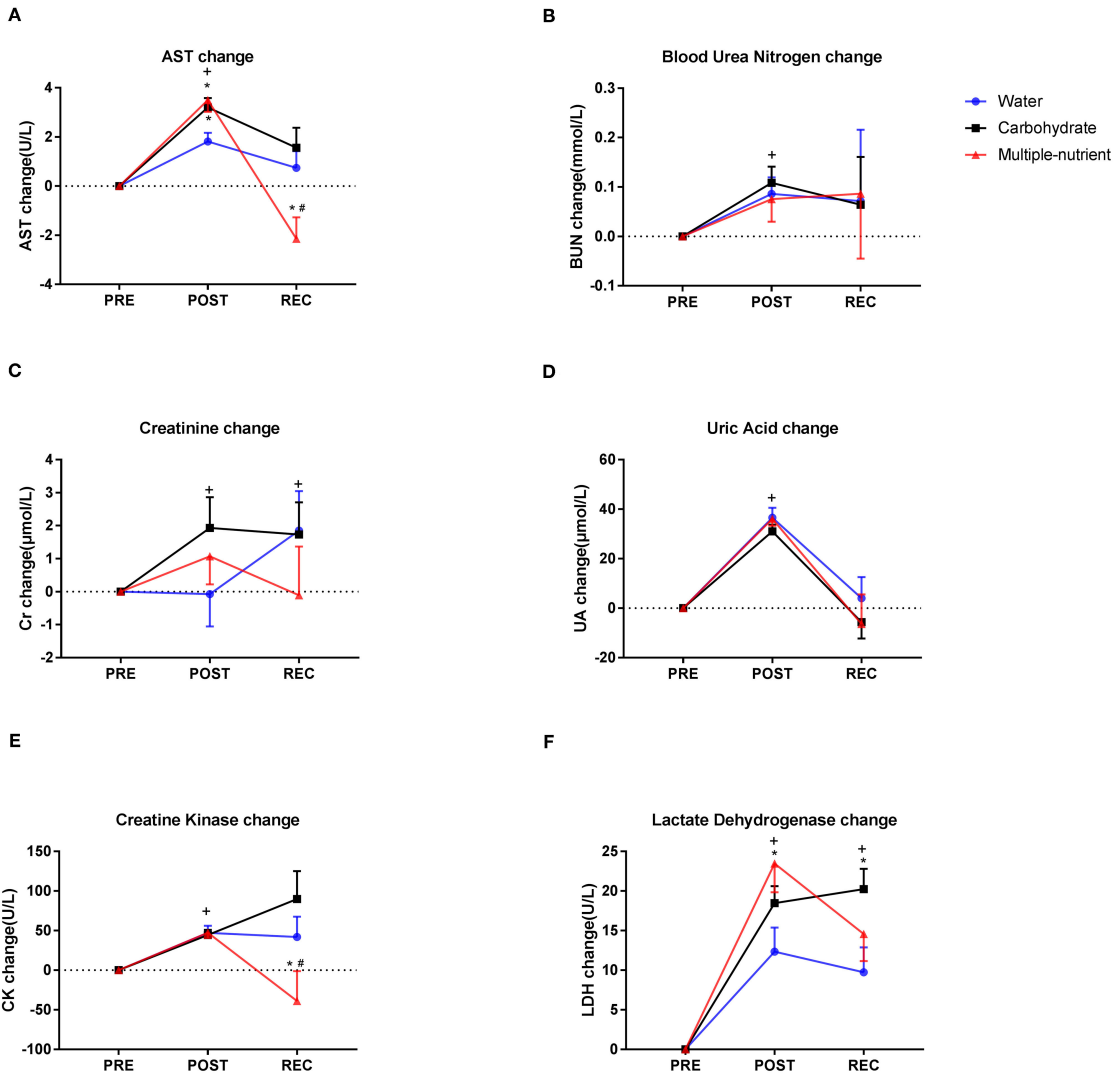
Changes in biochemical parameters in the short-term supplement trial. Changes in AST **(A)**, BUN **(B)**, Cr **(C)**, UA **(D)**, CK **(E)**, and LDH **(F)** levels in different experimental groups. Values are mean ± SEM. PRE, prior running test; POST, immediately after running test; REC, 24 h after running test. Change at POST equals POST minus PRE, change at REC equals REC minus PRE. *Indicates difference compared with water, *p* < 0.05. ^#^Indicates a difference compared with glucose, *p* < 0.05. +Indicates difference compared with PRE, *p* < 0.05.

Figures 3A–F portrait the changes in biochemical parameters in the short-term supplement trial. Levels of AST significantly increased immediately at POST, and the elevation extent was significantly larger in the M group and G group than in the W group (*p* = 0.010). The elevation of AST dropped more quickly in the M group than in any of the other two groups at REC (*p* = 0.012). M group significantly reduced AST elevation compared with carbohydrates at REC in the pilot trial (Supplementary Figure 2A).

For markers of renal function in the pilot trial, the elevation extent of BUN was larger in the W group than in the G group and M group at POST (*p* < 0.01). Changes in Cr and UA levels in the M group were the smallest compared with the other two groups at REC (*p* < 0.01 for both Cr and UA, (Supplementary Figures 2B–D). Similar trends, although not significant, were found in the short-term supplement trial in terms of BUN, Cr, and UA changes (Figures 3B–D).

Creatine kinase changes were equal among the groups at POST, while it was statistically lower in the M group than in either the G group or W group at REC (*p* = 0.045, Figure 3E). Figure 3F indicates a greater LDH elevation at POST in the M group than in the W group (*p* = 0.038). However, LDH elevation was found to be greater in the G group than in either the W group or the M group (*p* = 0.033 at REC). Similar trends, although not significant, were found in the pilot trial, in which the elevation of CK and LDH tended to be greater in the G group than in the other two groups (Supplementary Figures 2E,F).

### Multiple-Nutrient Supplementation Reduced Inflammatory and Oxidative Cytokines

**Figure 4:**
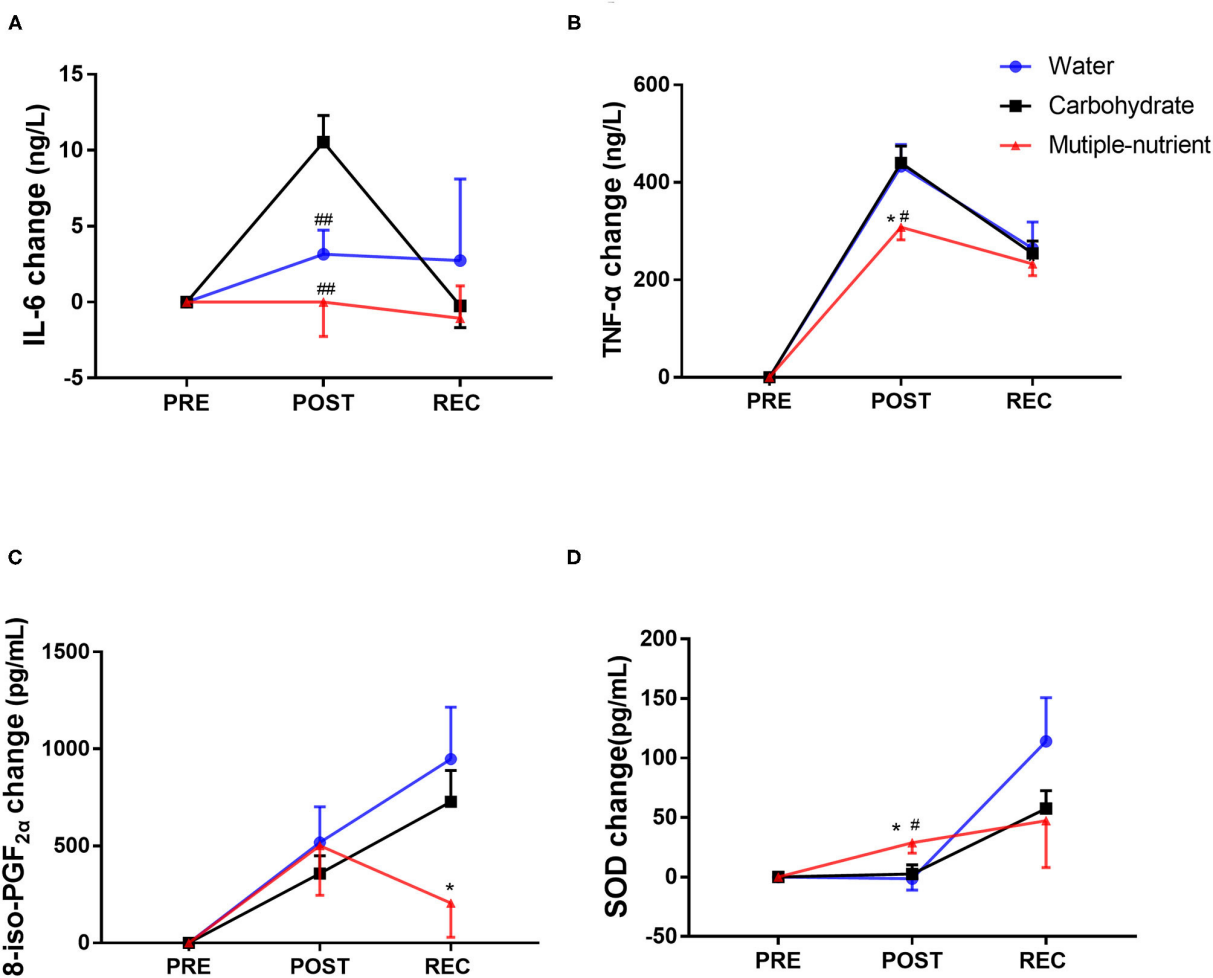
Changes in inflammatory factors and oxidative stress parameters in the short-term supplement trial. Changes in (interleukin 6) IL-6 **(A)**, tumour necrosis factor-α (TNF-α) **(B)**, 8-iso-prostaglandin F (2alpha) (8-iso-PGF2α) **(C)** and superoxide dismutase (SOD) **(D)** levels in different experimental groups. Values are mean ± SEM. PRE, prior running test; POST, immediately after running test; REC, 24 h after running test. Change at POST equals POST minus PRE, change at REC equals REC minus PRE. *Indicates difference compared with water, *p* < 0.05. ^#^Indicates a difference compared with glucose, *p* < 0.05, ^##^Indicates a difference compared with glucose, *p* < 0.01.

Figure 4 presents parameters reflecting inflammatory reactions and oxidative stress. Inflammation factors were elevated most the highest at POST and decreased at REC. Changes in IL-6 were significantly smaller in the M group and W group at POST than in the C group (*p* = 0.001, Figure 4A). Simultaneously, the changes in TNF-α in the M group were smaller than those in any of the other two groups at POST (*p* = 0.015, Figure 4B). 8-iso-PGF2α was increased immediately after the running test, and no significant difference was found among the groups at POST. However, it continued to increase 24 h after the running test except in the M group and then led to the smallest elevation in the M group at REC (*p* = 0.036, Figure 4C). Moreover, the increase in SOD was the largest in the M group among the three groups at POST (*p* = 0.033, Figure 4D). No significant difference was found regarding inflammatory factors in the single-shot supplement trial (Supplementary Figure 3).

### Influences of Multiple-Nutrient Supplement on Running Performance and Its Side Effects

**Figure 5:**
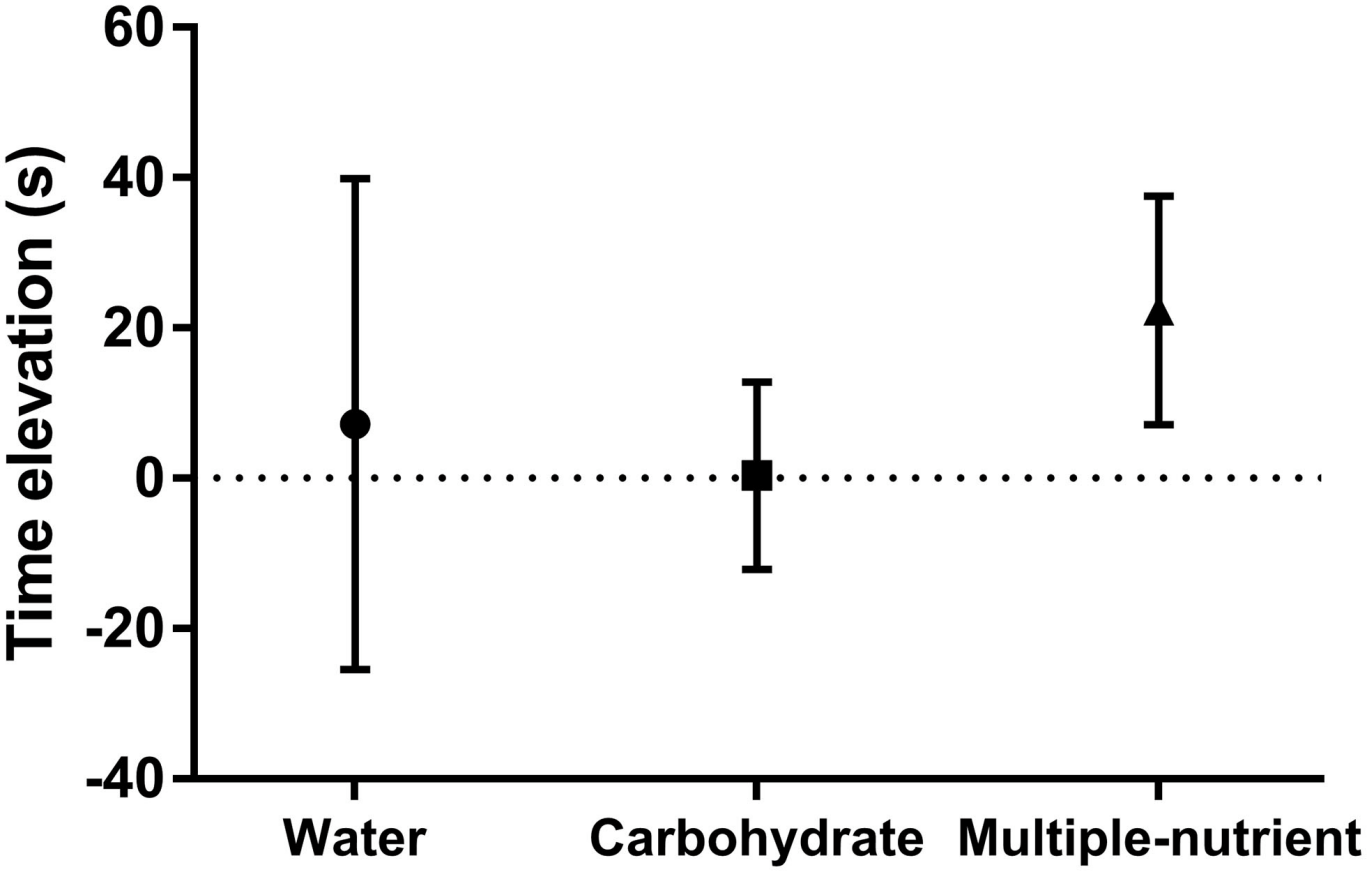
Elevation of running time after 7 days of supplementation in the short-term supplement trial. Values are mean ± SEM.

Figure 5 presents the time elevation of the running test after a 7-day supplement. There was no significant difference among the groups at baseline. The running time was slightly shorter after 7 days of supplementation, although the difference was not significant.

No significant difference was noted in terms of RPE in either experiment (presented in Supplementary Figure 4). No serious gastrointestinal symptoms, such as omission or pain, were found during these two trials.

## Discussion

In this study, faster recovery from muscle damage and compromised liver and renal function were found with supplementation with multiple nutrients after exercising in hot conditions. A pilot trial and a randomised controlled trial were constructed in the present study. The change in BUN at POST and changes in AST, Cr, and UA at REC significantly decreased after a single-shot supplement in the pilot trial. Data obtained in the short-term supplement trial corroborate the findings in the pilot trial. Multiple-nutrient supplementation significantly decreased changes in AST, CK, and LDH in the REC after a 7-day supplement. Meanwhile, a tendency to suppress the elevation of Cr and UA was found.

Either intense physical activity or heat exposure depresses glomerular filtration and renal plasma flow, thus leading to retention of UA and Cr (33). Cr was produced from the breakdown of creatine phosphokinase in muscle. Nucleic acids released from damaged muscle increased UA generation. However, multiple-nutrient supplementation attenuated kidney excretion suppression and accelerated the clearance of metabolites in this study. Additionally, dehydration can lead to the elevation of Cr and BUN. The least elevation of multiple-nutrient supplement of Cr indicates better hydration in the pilot trial. It has been proven that heat- and exercise-induced muscle damage, inflammatory responses, and oxidative stress can cause acute kidney injury (34, 35). In the current study, multiple-nutrient supplementation may attenuate these stresses to protect the kidney.

Similarly, intense exercise under heat can induce muscle damage, liver hypoperfusion, and ischaemia. Then, the repair signal was activated, and the migration of circulating blood neutrophils and monocytes toward the damaged site was triggered. Immune cells promote the degradation of cellular debris and phagocytosis by producing free radicals, which leads to secondary damage and increased permeability of the membrane. As a result, increased levels of AST, CK, and LDH are released into the bloodstream (36). Our results support previous findings that the levels of AST, CK, and LDH increased after exercise (37, 38).

In the present study, we noticed a greater AST elevation in the multiple-nutrient supplement group immediately after the running test. However, it decreased rapidly during recovery, which means that its transient elevation could be physiological. Significantly greater AST, CK, and LDH activities for glucose and water supplementation during recovery suggest greater membrane damage caused by lipolytic enzymes activated by pro-inflammatory cytokines (36, 39).

Antioxidants were generally used in sports supplements. Recently, a meta-analysis concluded that vitamin C attenuated oxidative stress and the inflammatory response (40). Beyond that, studies found that taurine ingestion for 7 days to 8 weeks decreased oxidative stress in both cyclists and triathletes (41, 42). Previous studies found that antioxidants could protect the liver from exercise-induced damage by modulating the mRNA expression of both inflammation-related and oxidative-related signal transduction pathways (43, 44). Similarly, a greater elevation of SOD after the running test and a smaller change in 8-iso-PGF2α during recovery suggest that multiple-nutrient supplementation upregulated plasma antioxidant potential and helped prevent lipid peroxidation in this study. Simultaneously, greater elevations in inflammatory factors were detected after the running test in the carbohydrate group and water group than in the multiple-nutrient supplement group in our study. Therefore, it can be postulated that anti-inflammatory and antioxidative ingredients attenuate secondary damage to healthy myocytes.

Moreover, additional vitamin K2 was added to the multiple-nutrient supplement in the short-term supplement trial. Vitamin K2 (MK-7) was formerly used to prevent bone fracture. More recently, MK-7 has been found to be a bioactive compound that promotes ATP production by increasing the efficiency of the electronic transportable chain. Moreover, vitamin K2 could prevent inflammation and ROS accumulation without the risk of negative side effects or overdosing (45, 46). However, there is still a dearth of research focusing on its effect on physical exercise under heat. In the current study, an extra effect of decreased changes in CK, LDH, IL-6, TNF-α, and 8-iso-PGF2α during recovery was found, as expected, which indicated enhanced membrane integrity and reduced leakage of enzymes caused by secondary damage.

Furthermore, we found that serum glucose increased POSTs in both the single-shot supplement trial and short-term supplement trial. Similar results can be found from the former studies that exercise after fasting overnight does not necessarily lead to a drop in glucose concentration (47). This may be due to the breakdown of muscle glycogen during intense activities.

## Strengths and Limitations

A large number of subjects were enrolled in this study. The findings of the short-term supplement trial can further confirm those in the pilot trial. However, there are still some limitations to this study. First, this study consisted of two single-blinded trials, which could introduce potential bias. However, this allowed us to respond to emergencies and gastrointestinal symptoms, for example, in time. Moreover, we managed to control for potential bias while conducting these two trials. Participants were numbered randomly, and all data collection was performed according to their number. Only researchers who distributed beverages knew the grouping, and those who helped collect and analyse data did not. These two trials further validated each other. Although we managed to be unbiased, we still do not think that they were enough to be called as double-blinded trials. Second, men are more likely to engage in occupations that need to perform physical activity in hot environments. Current research has not focused on whether multiple-nutrient supplementation is equally effective in women. Finally, we found the effect of multiple-nutrient supplementation on attenuating the elevation of CK and LDH in the second trial but not in the pilot trial. Whether the benefit is due to the longer period supplementation or the extra addition of vitamin K2 is not explored in this study. Further studies are warranted to investigate the exact effect and mechanism behind this.

## Conclusions

The current study found that multiple nutrient supplements promoted the recovery of muscle damage, liver function, and kidney function, probably by reducing secondary damage and accelerating the clearance of metabolic products after exercising under heat. This study will provide evidence for further nutritional recommendations that instruct workers and athletes who engage in heavy physical activities under heat to recover in a timely manner.

## Data Availability Statement

The raw data supporting the conclusions of this article will be made available by the authors, without undue reservation.

## Ethics Statement

The studies involving human participants were reviewed and approved by the 962nd Hospital of the PLA Joint Logistic Support Force Ethics Committee and Harbin Medical University Ethics Committee. The patients/participants provided their written informed consent to participate in this study.

## Author Contributions

YL, XuW, and CW contributed to conceiving and designing this study. CW, SZ, YZ, WG, SL, and BL performed the literature review, carried out the experiment, and collected data. YZ analysed and interpreted the data. CW and SZ wrote the first manuscript. WG, SK, and XuaW revised the manuscript. The final version of this manuscript was checked and approved by all the listed co-authors. All authors contributed to the article and approved the submitted version.

## Funding

This study was supported by PLA logistic research project (NO. AWS16J023).

## Conflict of Interest

The authors declare that the research was conducted in the absence of any commercial or financial relationships that could be construed as a potential conflict of interest.

## Publisher's Note

All claims expressed in this article are solely those of the authors and do not necessarily represent those of their affiliated organizations, or those of the publisher, the editors and the reviewers. Any product that may be evaluated in this article, or claim that may be made by its manufacturer, is not guaranteed or endorsed by the publisher.

## Supplementary Material

Supplementary Figure 1 Fatigue-related parameters of each group in the single-shot supplement trial. Glucose levels before and after running test **(A)**. Individual response of each group **(B)**. Change in lactic acid levels at different time points **(C)**. Values are mean ± SEM. PRE, prior running test; POST, immediately after running test. ^**^Indicates *p* < 0.01. +Indicates difference compared with PRE, *p* < 0.05.

Supplementary Figure 2 Changes in AST **(A)**, BUN **(B)**, Cr **(C)**, UA **(D)**, CK **(E)**, and LDH **(F)** levels in the pilot trial. Values are mean± SEM. PRE-prior running test; POST-immediately after running test; REC-24 hours after running test. Change at POST equals POST minus PRE, change at REC equals REC minus PRE. ^*^Indicates a difference compared with water, *p* < 0.05, ^**^indicates a difference compared with water, *p* < 0.01. #Indicates a difference compared with glucose, *p* < 0.05. ++Indicates difference compared with PRE, *p* < 0.01.

Supplementary Figure 3 Concentrations of TNF-α **(A)** and IL-6 **(B)** before and immediately after the running test. Values are mean± SEM. PRE-prior running test; POST-immediately after running test. ^**^Indicates *p* < 0.01.

Supplementary Figure 4 Rating of perceived exertion (RPE) of participants immediately after running the test in the pilot trial **(A)** and short-term supplement trial **(B)**. Values are mean± SEM.

Supplementary Table 1 Anthropometric characteristics of participants in the single-shot supplement trial at baseline. Data are presented as the means ± SEM. BMI, body mass index.

Supplementary Table 2 Biochemical parameters of participants in the single-shot supplement trial at baseline. Data are presented as the means ± SEM.

## Abbreviations

ROS: Reactive oxygen species
RPE: Rating of perceived exertion
PRE: Before running test
POST: Immediately after running test
REC: 24 h after running test
AST: Aspartate aminotransferase
ALT: Alanine aminotransferase
GLU, Glucose, BUN: Blood urea nitrogen
Cr: Creatinine
UA: Uric acid
CK: Creatine kinase
LDH: Lactate dehydrogenase
Na: Sodium
K: Potassium
IL-6: Interleukin-6
TNF-α: Tumour necrosis factor-α
8-iso-PGF2α: 8-iso-prostaglandin F(2alpha)
SOD: Superoxide dismutase.
