# Supplementary figures and images for: Effect of Multiple-Nutrient Supplement on Muscle Damage, Liver, and Kidney Function After Exercising Under Heat: Based on a Pilot Study and a Randomised Controlled Trial
Source: Front Nutr. 2021 Dec 23;8:740741. doi: 10.3389/fnut.2021.740741 (PMC8733564; doi:10.3389/fnut.2021.740741)

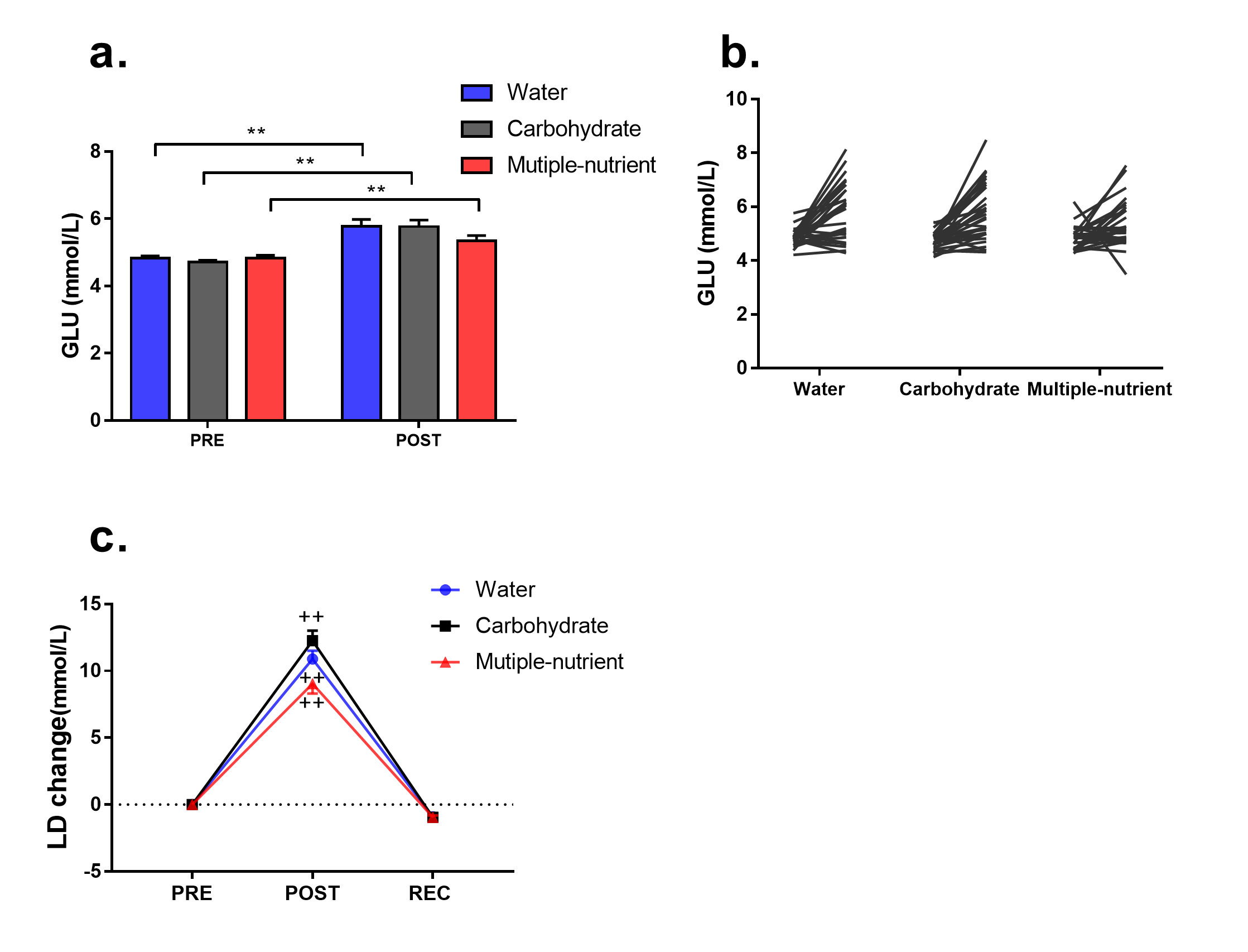

Supplement: Supplementary Figure 1 — Fatigue-related parameters of each group in the single-shot supplement trial. Glucose levels before and after running test (A). Individual response of each group (B). Change in lactic acid levels at different time points (C). Values are mean ± SEM. PRE, prior running test; POST, immediately after running test. **Indicates p < 0.01. +Indicates difference compared with PRE, p < 0.05. [file Image_1.tif]

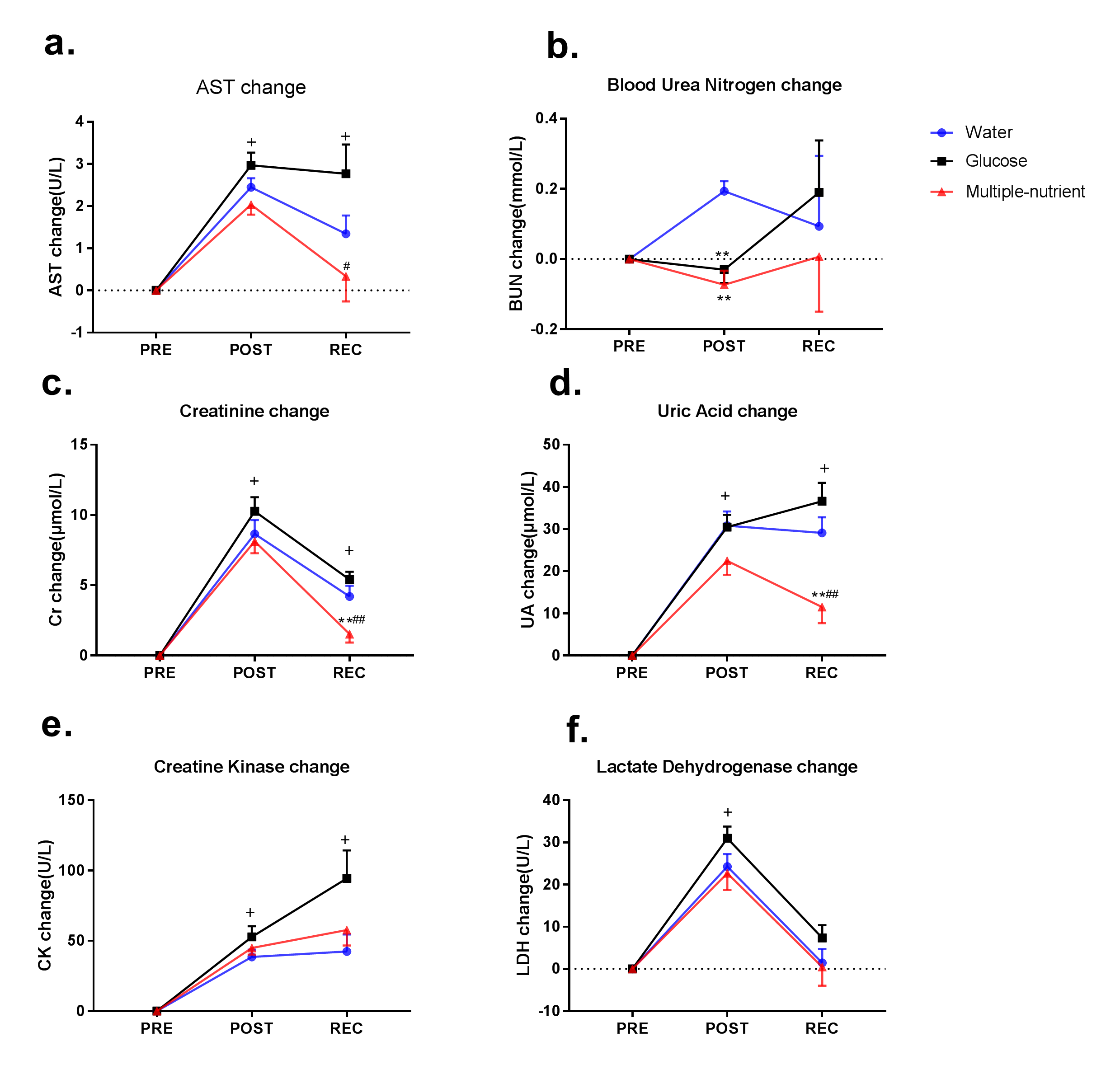

Supplement: Supplementary Figure 2 — Changes in AST (A), BUN (B), Cr (C), UA (D), CK (E), and LDH (F) levels in the pilot trial. Values are mean± SEM. PRE-prior running test; POST-immediately after running test; REC-24 hours after running test. Change at POST equals POST minus PRE, change at REC equals REC minus PRE. *Indicates a difference compared with water, p < 0.05, **indicates a difference compared with water, p < 0.01. #Indicates a difference compared with glucose, p < 0.05. ++Indicates difference compared with PRE, p < 0.01. [file Image_2.tif]

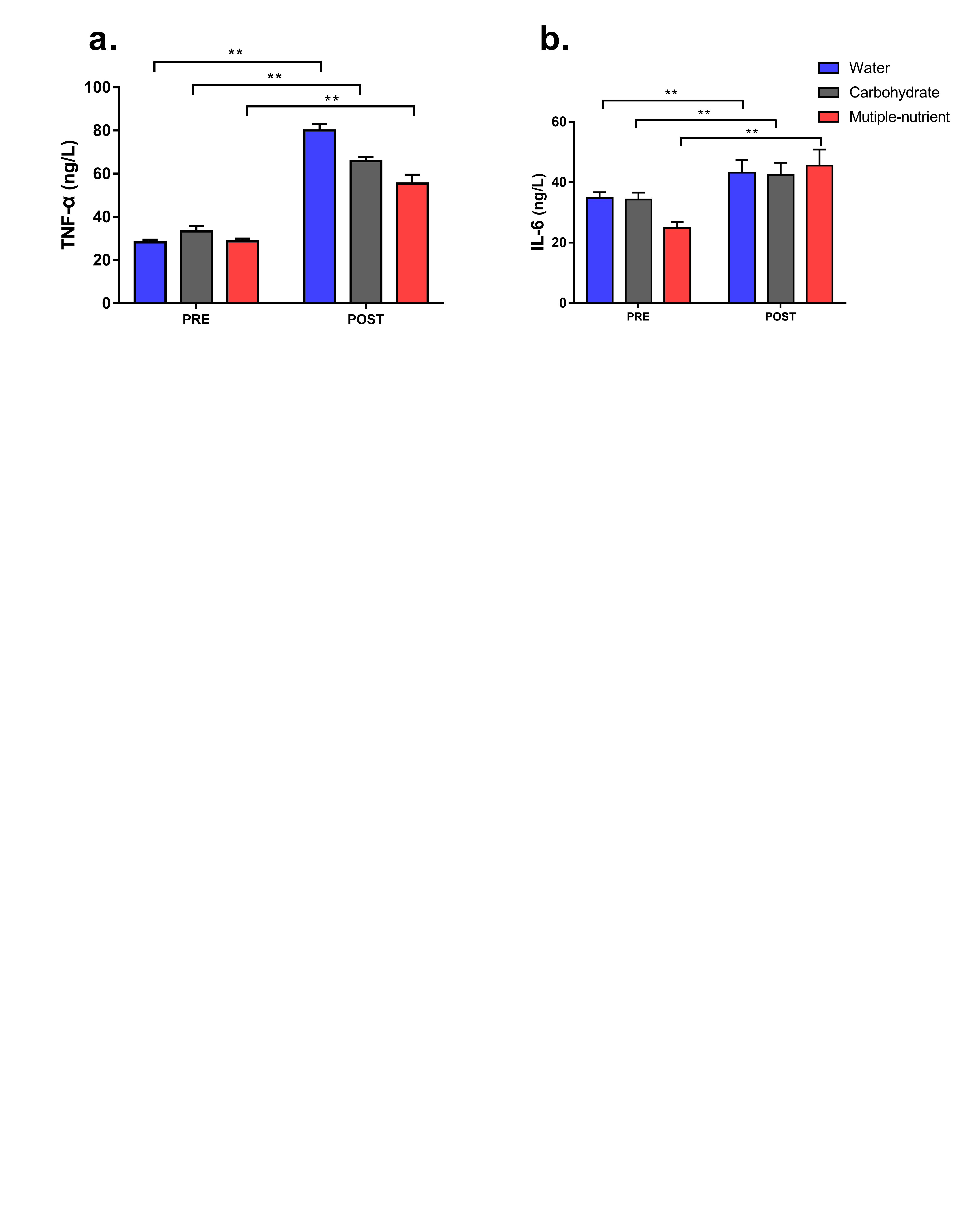

Supplement: Supplementary Figure 3 — Concentrations of TNF-α (A) and IL-6 (B) before and immediately after the running test. Values are mean± SEM. PRE-prior running test; POST-immediately after running test. **Indicates p < 0.01. [file Image_3.tif]

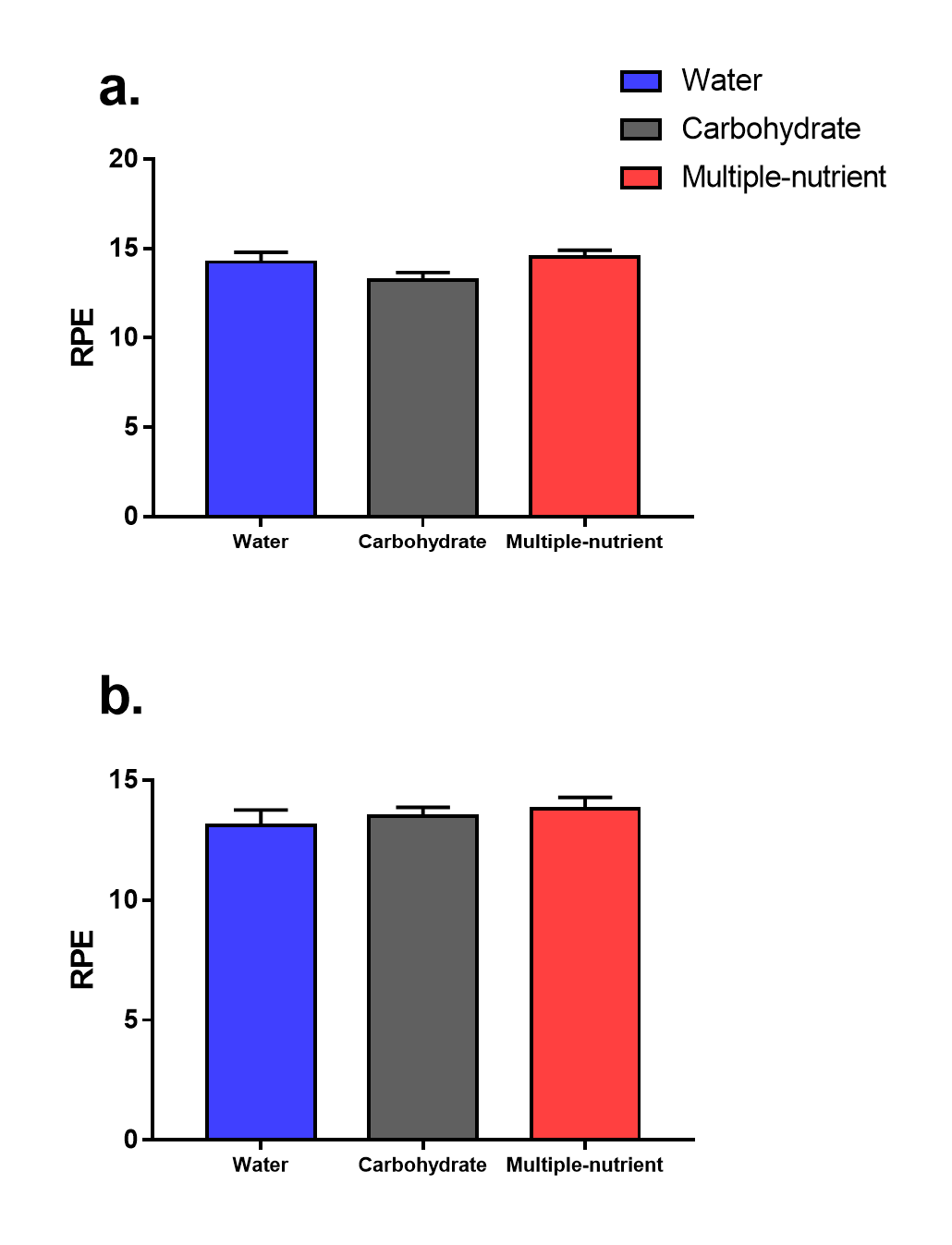

Supplement: Supplementary Figure 4 — Rating of perceived exertion (RPE) of participants immediately after running the test in the pilot trial (A) and short-term supplement trial (B). Values are mean± SEM. [file Image_4.tif]
