# Supplementary material for: Effect of Multiple-Nutrient Supplement on Muscle Damage, Liver, and Kidney Function After Exercising Under Heat: Based on a Pilot Study and a Randomised Controlled Trial
Source: Front Nutr. 2021 Dec 23;8:740741. doi: 10.3389/fnut.2021.740741 (PMC8733564; doi:10.3389/fnut.2021.740741)
Supplement: Supplementary Table 1 — Anthropometric characteristics of participants in the single-shot supplement trial at baseline. Data are presented as the means ± SEM. BMI, body mass index. [file Table_1.docx]

**Supplementary Table 1** Anthropometric characteristics of participants in single-shot supplement trial at baseline

|  | Water group | Carbohydrate group | Supplement group | *p* |
| --- | --- | --- | --- | --- |
| N | 29 | 30 | 30 |  |
| Age (y) | 22.78±0.54 | 23.28±0.59 | 22.51±0.51 | 0.608 |
| Length of work (y) | 4.00±0.36 | 4.70±0.60 | 4.20±0.45 | 0.937 |
| Height (cm) | 172.55±1.12 | 173.42±1.10 | 173.77±0.81 | 0.528 |
| Weight (kg) | 66.37±1.49 | 66.19±1.57 | 66.88±1.43 | 0.916 |
| Waist (cm) | 72.49±2.71 | 75.00±1.34 | 75.40±1.31 | 0.988 |
| Body fat percentage (%) | 17.58±0.90 | 17.71±0.89 | 16.82±0.93 | 0.755 |
| BMI(kg/m^2^) | 22.26±0.40 | 21.97±0.40 | 22.15±0.45 | 0.879 |
| Systolic pressure (mmHg) | 128.55±2.90 | 127.23±2.05 | 129.67±2.02 | 0.763 |
| Diastolic pressure (mmHg) | 70.93±1.96 | 71.73±1.74 | 71.63±1.50 | 0.939 |
| Heart rate (bpm) | 71.76±1.87 | 71.73±1.83 | 69.80±1.50 | 0.659 |

Data are presented as means± standard error of the mean (SEM). BMI-body mass index.
