# Supplementary material for: Effect of Multiple-Nutrient Supplement on Muscle Damage, Liver, and Kidney Function After Exercising Under Heat: Based on a Pilot Study and a Randomised Controlled Trial
Source: Front Nutr. 2021 Dec 23;8:740741. doi: 10.3389/fnut.2021.740741 (PMC8733564; doi:10.3389/fnut.2021.740741)
Supplement: Supplementary Table 2 — Biochemical parameters of participants in the single-shot supplement trial at baseline. Data are presented as the means ± SEM. [file Table_2.docx]

**Supplementary Table 2** Biochemical parameters of participants in single-shot supplement trial at baseline

|  | Water group | Carbohydrate group | Supplement group | *p* |  |
| --- | --- | --- | --- | --- | --- |
|  |  |  |  |  |  |
| ALT（U/L） | 20.52±2.49 | 18.70±2.07 | 20.50±3.55 | 0.868 |  |
| AST（U/L） | 22.86±0.86 | 21.97±0.87 | 21.93±1.34 | 0.781 |  |
| AST/ALT | 1.31±0.08 | 1.34±0.07 | 1.35±0.09 | 0.916 |  |
| GLU (mmol/L) | 4.84±0.06 | 4.71±0.06 | 4.84±0.08 | 0.302 |  |
| BUN (mmol/L) | 6.00±0.21 | 5.36±0.19 | 5.65±0.19 | 0.076 |  |
| CREA (μmol/L) | 70.03±1.44 | 68.70±1.56 | 68.97±1.36 | 0.793 |  |
| UA (μmol/L) | 377.25±14.20 | 388.47±13.14 | 378.67±10.20 | 0.790 |  |
| TG (mmol/L) | 0.91±0.06 | 0.97±0.09 | 1.12±0.08 | 0.116 |  |
| CK（U/L） | 169.69±20.08 | 153.50±11.49 | 145.63±10.38 | 0.495 |  |
| LDH（U/L） | 173.24±4.62 | 169.10±4.46 | 167.53±6.37 | 0.731 |  |

Data are presented as means± standard error of the mean (SEM).
